# Inhibition of Carotenoid Biosynthesis by CRISPR/Cas9 Triggers Cell Wall Remodelling in Carrot

**DOI:** 10.3390/ijms22126516

**Published:** 2021-06-17

**Authors:** Tomasz Oleszkiewicz, Magdalena Klimek-Chodacka, Michał Kruczek, Kamila Godel-Jędrychowska, Katarzyna Sala, Anna Milewska-Hendel, Maciej Zubko, Ewa Kurczyńska, Yiping Qi, Rafal Baranski

**Affiliations:** 1 Department of Plant Biology and Biotechnology, Faculty of Biotechnology and Horticulture, University of Agriculture in Krakow, 31-425 Krakow, Poland; tomasz.oleszkiewicz@urk.edu.pl (T.O.); m.chodacka@urk.edu.pl (M.K.-C.); kruczek.michael@gmail.com (M.K.); 2 Institute of Biology, Biotechnology and Environmental Protection, Faculty of Natural Sciences, University of Silesia in Katowice, 40-032 Katowice, Poland; kamila.godel-jedrychowska@us.edu.pl (K.G.-J.); katarzyna.sala@us.edu.pl (K.S.); anna.milewska@us.edu.pl (A.M.-H.); ewa.kurczynska@us.edu.pl (E.K.); 3 Institute of Materials Engineering, Faculty of Science and Technology, University of Silesia, 41-500 Chorzów, Poland; maciej.zubko@us.edu.pl; 4 Department of Physics, Faculty of Science, University of Hradec Králové, 500 03 Hradec Králové, Czech Republic; 5 Department of Plant Science and Landscape Architecture, University of Maryland, College Park, MD 20742, USA; yiping@umd.edu

**Keywords:** arabinogalactan protein, callus, Cas9 protein, chromoplasts, Clustered Regularly Interspaced Short Palindromic Repeats, CRISPR, pectins, phytoene synthase, plastid ultrastructure

## Abstract

Recent data indicate that modifications to carotenoid biosynthesis pathway in plants alter the expression of genes affecting chemical composition of the cell wall. Phytoene synthase (PSY) is a rate limiting factor of carotenoid biosynthesis and it may exhibit species-specific and organ-specific roles determined by the presence of *psy* paralogous genes, the importance of which often remains unrevealed. Thus, the aim of this work was to elaborate the roles of two *psy* paralogs in a model system and to reveal biochemical changes in the cell wall of *psy* knockout mutants. For this purpose, Clustered Regularly Interspaced Short Palindromic Repeats (CRISPR) and CRISPR associated (Cas9) proteins (CRISPR/Cas9) vectors were introduced to carotenoid-rich carrot (*Daucus carota*) callus cells in order to induce mutations in the *psy1* and *psy2* genes. Gene sequencing, expression analysis, and carotenoid content analysis revealed that the *psy2* gene is critical for carotenoid biosynthesis in this model and its knockout blocks carotenogenesis. The *psy2* knockout also decreased the expression of the *psy1* paralog. Immunohistochemical staining of the *psy2* mutant cells showed altered composition of arabinogalactan proteins, pectins, and extensins in the mutant cell walls. In particular, low-methylesterified pectins were abundantly present in the cell walls of carotenoid-rich callus in contrast to the carotenoid-free *psy2* mutant. Transmission electron microscopy revealed altered plastid transition to amyloplasts instead of chromoplasts. The results demonstrate for the first time that the inhibited biosynthesis of carotenoids triggers the cell wall remodelling.

## 1. Introduction

Carotenoids are a vast group of compounds that widely exist in nature. In plants, they play crucial roles in photosynthetic systems and they are precursors of phytohormones [1]. Beneficial effects of carotenoids on human health are extensive. For example, β-carotene and α-carotene have provitamin A activity, and others are beneficial for treatment of age-related macular degeneration or in prevention of cardiovascular disorders and cancer [2]. The biosynthesis and accumulation of carotenoids in plants occurs in plastids and is regulated by developmental and environmental factors [3,4]. Unlike in green tissues where carotenoids are components of chloroplasts, in non-green tissues they accumulate in chromoplasts that develop from leucoplasts and amyloplasts [5]. The chromoplast ultrastructure is complex due to the transition from one plastid type to another. Carotenoids are sequestered mainly as membrane embedded crystals or as lipid droplets enclosed in plastoglobuli [6].

Several signalling compounds are products of carotenoid degradation such as abscisic acid (ABA) and strigolactones, which regulate plant processes including germination, plant architecture, recognition of arbuscular mycorrhizal fungi, and response to stresses [7,8]. In recent years, there has been an increasing amount of data suggesting a relationship between the biosynthesis of carotenoids and the chemical composition of the cell wall. The available data indicate that the overexpression or disturbance in the expression of carotenoid genes affects, among others, the expression of genes that take part and regulate the biosynthesis of cell wall components [9,10,11]. Moreover, strigolactones may participate in the secondary wall architecture remodelling [12]. However, to our best knowledge, no direct evidence has been shown linking carotenoid metabolism and primary cell wall remodelling due to changes in wall components such as pectins, arabinogalactan proteins (AGPs), or extensins.

The main carotenoid biosynthesis pathway in plants has been well described [13,14]. The biosynthesis of an acyclic 15-*cis*-phytoene, the first carotene in the pathway, is catalysed in a two-step reaction by phytoene synthase (PSY). Generation of PSY is a rate-limiting step of carotenogenesis [15]. Various PSY isoforms have been described, but their number and activity differ among plant species. Only one *psy* gene was found in Arabidopsis [13] while multiple *psy* genes exist in many other species. In tobacco, PSY1 and PSY2 proteins have some functional redundancy and are mainly expressed in the aerial plant parts [11]. Both *psy1* and *psy2* genes were strongly expressed in potato tubers accumulating carotenoids [16]. However, they may also show organ-specific activities. In tomato, high expression was found for *psy1* in fruits [17], *psy2* in leaves [18], and *psy3* in roots [19]. In melon, *psy1* is expressed in leaves and fruits, and *psy2* in roots [20]. Furthermore, environmental conditions may modulate *psy* expression [3,4]. Phytochrome and cytochrome receptors regulate *psy* transcription in light exposed organs [21]. In carrot, two paralogous genes, *psy1* and *psy2*, have been found to code functional enzymes [22,23], but their differential role and regulation have not been fully investigated. According to the results of phylogenetic analysis, carrot *psy1* has been grouped with the Eudicot *psy2* clade, while *psy2,* being close to *psy* in *Solanum* species, has been grouped with Eudicot *psy1* [19]. The transcript level in carrot cultivars was up to three times higher for *psy1* than for *psy2* [22]. While the expression of both genes positively correlates with carotenoid contents, the *psy1* expression is upregulated by light in leaves, suggesting PSY1 plays more important role in green tissues than in the root. The expression of *psy2* in the root remains unaffected by light while it is repressed in the leaves [24,25,26]. Besides such organ specificity, *psy2* is also differentially expressed in xylem and phloem tissues in the carrot root [27] and the *psy2* expression is further induced under salt stress through ABA signalling pathway [28]. However, in white and orange carrot roots incomplete correlations between *psy* transcript levels and carotenoid contents were found [25,29]. The expression of both genes occurs in every part of the carrot plant, but it is not known if they complement each other or the carotenogenesis can be realised when either of them is inactive.

Callus obtained and cultured on mineral medium in vitro has been successfully utilized for studying carotenoid metabolism and accumulation [30,31]. However, carrot callus developing from root explants are poor in carotenoids, even if the roots are rich in carotenoids [32,33]. For this reason, the usefulness of such material for research on carotenogenesis is questionable. Recently, we have obtained and characterised a dark orange (d-o) carrot callus containing similar amounts of carotenoids as the root of orange carrot from which it was developed [34]. It differs from callus of low carotenoid content at morphological and ultrastructural levels, with visible carotene crystals in the cells. Several carotenogenesis related genes were expressed at similar levels as in the root. Both *psy* paralogs were expressed in d-o callus, although the expression level of *psy2* was much higher, resembling the relationship observed in the root. This model d-o callus is suitable for elucidating genetic background of carotenoid biosynthesis and sequestration as it has already been demonstrated in structural studies of carotene crystals [35,36].

The aim of this work is to elaborate the roles of *psy* paralogs in carrot and to verify whether carotenogenesis may occur if either *psy1* or *psy2* are non-functional. By designing and introducing Clustered Regularly Interspaced Short Palindromic Repeats (CRISPR) and CRISPR associated (Cas9) proteins (CRISPR/Cas9) vectors we generated functional mutations in *psy1* and *psy2* genes and demonstrate their effect on carotenoids accumulation and mutual regulation of their expression. Furthermore, having a *psy* functional mutant we show changes in callus histology, and plastid ultrastructure, in particular the development of the amyloplasts instead of chromoplasts. Moreover, we show for the first time, that the inhibited biosynthesis of carotenoids is accompanied by the cell wall remodelling that is manifested by changes in the compositions of AGPs, pectins, and extensins in the wall.

## 2. Results

### 2.1. Generating of psy1 and psy2 Mutants by CRISPR/Cas9

**Figure 1:**
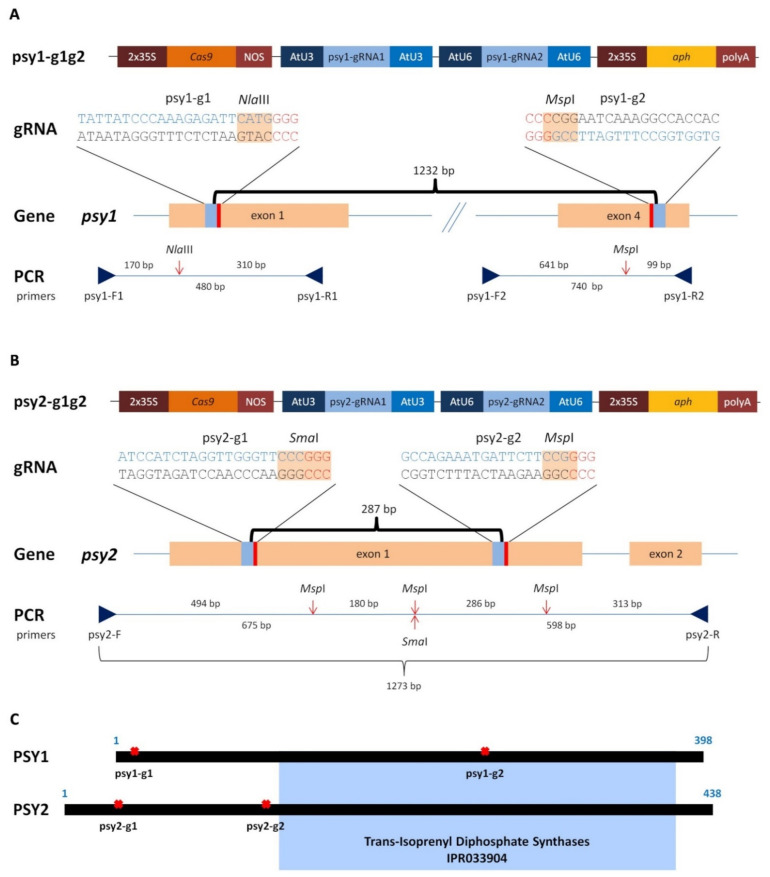
A scheme of Cas9 vectors (psy1-g1g2 and psy2-g1g2), each with two gRNAs (g1 and g2) targeting *psy* carrot genes and the PCR amplified fragments with primer names, restriction sites, and their lengths for (**A**) *psy1* and (**B**) *psy2*. (**C**) A linear scheme of PSY protein with the conserved domain marked in blue. The restriction sites are marked by red arrows and the gRNA:Cas9 target sites are marked by red crosses.

**Figure 2:**
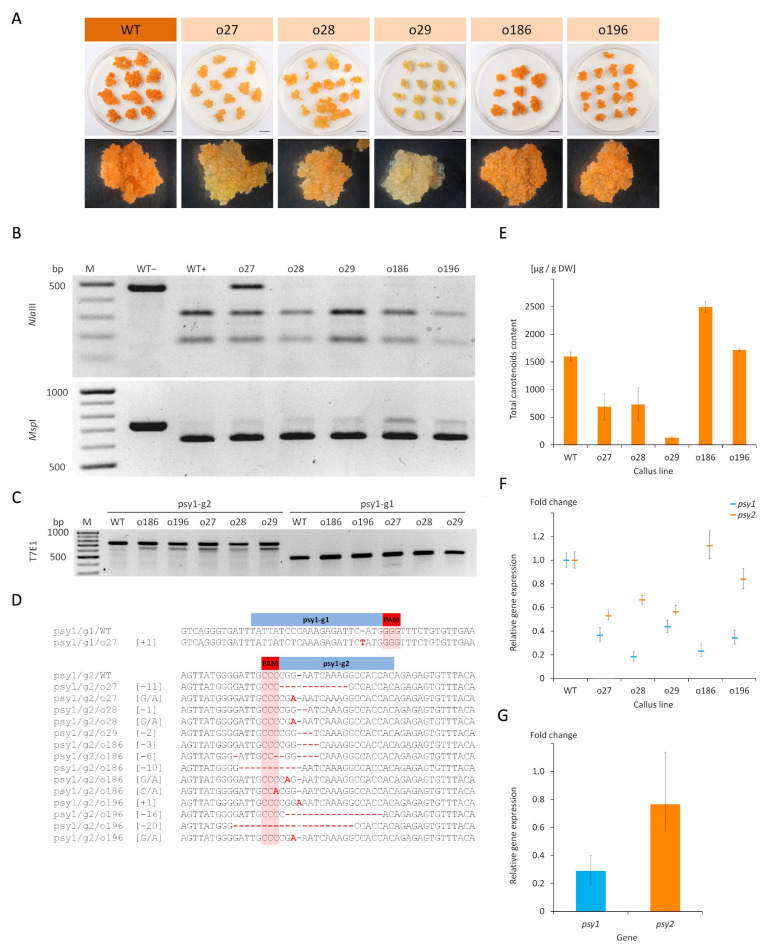
Carrot callus lines selected after transformation using the psy1-g1g2 vector with gRNAs targeting the *psy1* gene. (**A**) Developing callus in vitro. Separation of PCR fragments after restriction with (**B**) specific endonucleases and (**C**) T7EI enzyme. (**D**) Results of Sanger sequencing of target sites in the *psy1* gene. (**E**) Total carotenoid content. Relative *psy1* and *psy2* gene expressions in (**F**) each callus line and (**G**) the mean relative expressions. Scale bars represent 1 cm; WT–wild type callus (not transformed control) before (–) and after (+) restriction; o27-o196–transgenic callus lines; M–GeneRuler DNA Ladder Mix (Thermo Scientific, Waltham, MA, USA) marker.

**Figure 3:**
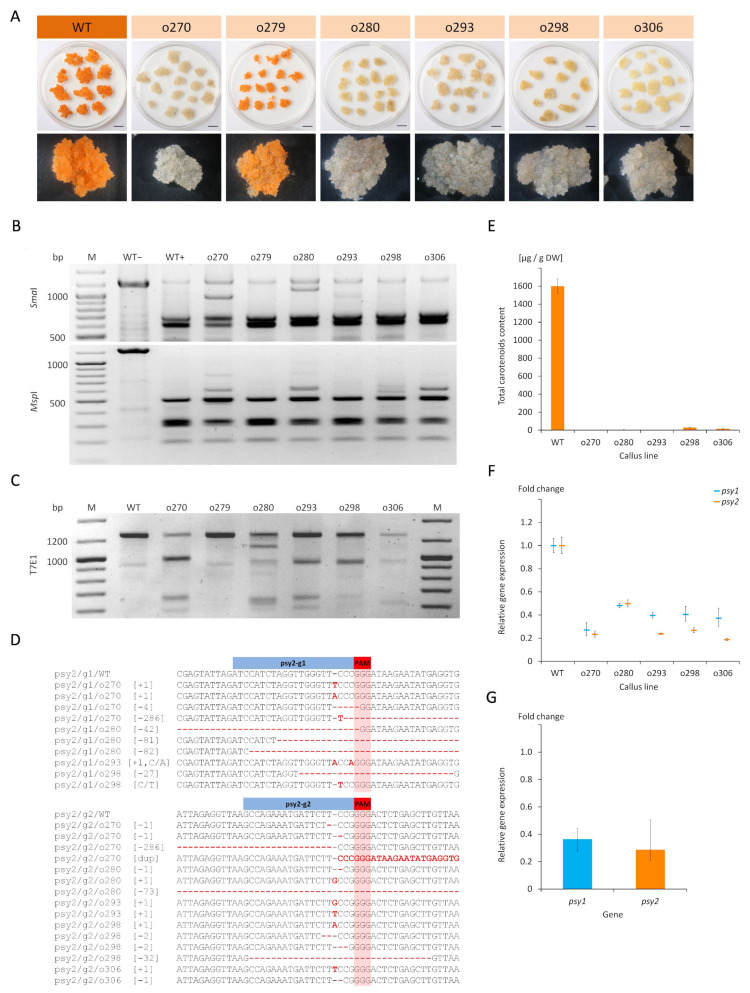
Carrot callus lines selected after transformation using the psy2-g1g2 vector with gRNAs targeting the *psy2* gene. (**A**) Developing callus in vitro. Separation of PCR fragments after restriction with (**B**) specific endonucleases and (**C**) T7EI enzyme. (**D**) Results of Sanger sequencing of target sites in the *psy2* gene. (**E**) Total carotenoid content. Relative *psy1* and *psy2* gene expressions in (**F**) each callus line and (**G**) the mean relative expressions. Scale bars represent 1 cm; WT−wild type callus (not transformed control) before (–) and after (+) restriction; o270-o306–transgenic callus lines; M—GeneRuler DNA Ladder Mix (Thermo Scientific) marker.

The role of two *psy* paralogs in the carotenoid biosynthesis was elucidated in the model callus system and for this purpose both genes were targeted during experiments aiming in the generation of knockout mutations. Two vectors, each with the Cas9 gene and a pair of gRNAs (namely g1 or g2), targeting either the *psy1* or *psy2* gene (Figure 1) were delivered to a dark-orange, carotenoid-rich carrot callus cells using *Agrobacterium*-mediated transformation. Callus incubated with *Agrobacterium* and transferred to a hygromycin selection medium developed new distinguishable cell clumps on the explants surface. They had mainly orange colour, but after subsequent transfers the differences in colour among callus lines became clearly visible. Those transformed with the psy1-g1g2 vector, to target the *psy1* gene, remained predominantly orange but varied from dark to pale orange or yellowish. Similar phenotype was observed for psy2-g1g2 callus, in which the *psy2* gene was targeted, but additionally, several white clumps developed. New white clumps were also developing on the surface of orange psy2-g1g2 callus in a prolonged culture which was not observed for psy1-g1g2 callus. The presence of *aph* and *Cas9* genes was confirmed in PCR. Hence, the developed callus lines were considered transgenic with introduced putatively active CRISPR/Cas9 editing system. Well grown calli were selected (Figure 2A and Figure 3A) for further characterisation regarding carotenoid content, expression of the *psy* genes and occurred mutations.

### 2.2. Characterisation of the psy1 Mutants

The developing callus lines after transformation with the psy1-g1g2 vector were characterised. Molecular analyses were performed to verify whether mutations were generated in the targeted *psy1* gene. The PCR amplification of DNA resulted in the expected products of either 480 bp or 740 bp depending on whether primers flanking the psy1-g1 or psy1-g2 target sites were used, respectively. The use of primer pair flanking both targets resulted in the expected 1495 bp product. The PCR products were then incubated with specific enzymes recognising restriction sites near protospacer adjacent motif (PAM) at each gRNA targets to reveal the occurrence of site mutations. All samples showed the restriction pattern of fragments whose sizes corresponded to those obtained for the wild type (WT), i.e., non-transgenic callus (Figure 2B).

Additionally, undigested products of various intensities were identified, indicating induced mutations by Cas9. The digestion of psy1-g1 amplicons using *Nla*III resulted in an intense band of undigested product for o27 line. The digestion of psy1-g2 amplicons using *Msp*I resulted in undigested products for all samples. T7EI assay was applied to reveal mutations that might also occur at sites other than those recognised by *Nla*III or *Msp*I. For WT, amplicons treated with T7EI remained intact and were visible as single band in the gel. When the psy1-g1 amplicons were treated, an additional faint band of shorter products indicating a mutation was obtained for o27 callus. For the psy1-g2 amplicons, all samples produced a single intense band of shorter products, which were not present in WT (Figure 2C). These results indicated the occurrence of mutations caused by Cas9 at the psy1-g2 target site or at both target sites in case of o27 line.

Sanger sequencing was further used to characterise the mutations and it confirmed a single nucleotide (T) insertion at the *Nla*III restriction site in the psy1-g1 region in o27 callus (Figure 2D). All psy1-g1g2 callus lines had mutations at the psy1-g2 region. They were single nucleotide (A) insertions, C/A and G/A substitutions, and deletions ranging from −1 to −20 nt (Figure 2D). Mutations occurred at the *Msp*I restriction site and either upstream or downstream this site. Single mutation was found for o29 callus while 2−5 variants were identified for the other callus lines. In silico translation revealed that the identified single nucleotide insertion in the psy1-g1 region resulted in a reading frame shift, generating a premature stop codon at the 26 amino acid (aa) position (Supplementary Materials, Figure S1A). Indel mutations in the psy1-g2 region resulted in a premature stop codon between 258 and 269 aa or, in case of −3 nt deletion, the deletion of one amino acid at the 250 position. Nucleotide substitutions mostly resulted in amino acid change at the 249 or 250 positions. Single silent mutation was found in o186 callus. Other nonsense mutations occurred in this line as well. Hence, amino acid sequence modifications were identified in all psy1-g1g2 callus lines.

Quantitative determination of carotenoids content was then performed to confirm that callus colour variation was related to the amounts of these pigments and to the induced mutations. WT callus contained carotenoids in the amount of 1599 µg/g DW which consisted of predominantly of β- and α-carotene, and traceable amounts of xanthophylls. In the transgenic psy1-g1g2 callus, the total carotenoids content highly varied among lines (*p* < 0.001) and ranged from 131 to 2493 µg/g DW (Figure 2E). Lines o27 and o28 had reduced amounts of carotenoids by a half (43% and 46% of WT, respectively) while the o29 line had very low amounts at the level of 8% in comparison to WT. Lines o196 did not differ from WT and o186 had higher amounts of carotenoids than WT. The observed variation in callus colour from dark orange to pale orange or yellowish is thus well explained by the total carotenoid content. The different amounts of carotenoids among these transgenic lines indicate different gene knockout levels for *psy1*.

The *psy1* gene expression was considerably down-regulated (*p* = 0.002) in all callus lines and it was at the 0.19−0.46 level relative to WT (Figure 2F) with the mean of 0.29 (Figure 2G). At the same callus, the expression of *psy2* gene did not differ from WT (*p* = 0.117) with the mean relative level of 0.77 (Figure 2G). However, three lines (o27, o28, and o29) had a lower *psy2* expression than WT while for o186 and o196 the *psy2* expression remained unchanged (Figure 2(F)). There was no correlation between the *psy1* and *psy2* expressions (*r* = −0.265; *p* = 0.612). The carotenoids content did not correlate with the *psy1* expression (*r* = 0.05; *p* = 0.923) but highly correlated with the *psy2* expression (*r* = 0.94; *p* = 0.006). The consistent downregulation of *psy1*-edited lines suggests nonsense-mediated mRNA decay (NMD) may play a role in regulating *psy1* expression.

### 2.3. Characterisation of the psy2 Mutants

Analogously as for the *psy1* mutants, the psy2-g1g2 callus lines were characterised. The PCR amplification of DNA isolated from WT and psy2-g1g2 callus was done using primers flanking both gRNA targets and resulted in the expected 1273 bp products. Additionally, either shorter products of about 300 bp, corresponding to the 286 nt distance between psy2-g1 and psy2-g2 targets, or longer products of about 300 bp were identified for the o270 line, and the product shorter by about 80 bp was found for the o280 line. For WT callus, the digestion of PCR products with *Sma*I resulted in two expected cleavage products. The same reaction performed for the psy2-g1g2 lines revealed the presence of the same cleaved products and a fainter band of undigested amplicons (Figure 3B). Additionally, the above listed shorter PCR products by about 80 and 300 bp were identified for the o270, o280, and o293 lines. Digestion with *Msp*I resulted in new bands for o270, o280, o298, and o306, but not for WT. Hence, products other than those observed for WT were identified for all white callus lines. The T7EI assay resulted in complex band profiles differing among callus lines, indicating diverse mutations were introduced by Cas9. Additional products ranging from 300 to 1150 bp and not distinguishable in WT were identified for all white callus lines (Figure 3C). The band profile for orange o279 callus was the same for WT, regardless which endonuclease was used. Thus, the obtained results indicated that all white callus lines were mutants.

Sequencing of PCR products confirmed mutations in both gRNA target regions (Figure 3D). Most mutations were single nucleotide insertions (A, T, or G) and deletions, also C/A and C/T substitutions were found. Deletions were either short (−1 to −4 nt) or relatively long (−27 to −82 nt). Additionally, the deletion of the whole fragment spanning between PAMs at both gRNA targets (−286 nt) was found for o270 callus. In the same callus, a duplication of this fragment also occurred. For the o270 and o293 lines, the mutations at the psy2-g1 region resulted in the occurrence of premature stop codons 3−4 aa downstream the mutation sites (Supplementary Materials, Figure S1B). For the o280 and o298 lines, the −27 to −81 nt deletions resulted in the removal of peptide fragments of 9 to 27 aa, and for o270, the −86 nt deletion caused 95 aa fragment removal and reading frame shift. Mutations generated at the psy2-g2 region resulted in the premature stop codons at the locations close to the target site. Hence, all identified mutations considerably affect PSY2 aa sequence and are likely null.

In contrast to the carotenoid-rich WT callus, the psy2-g1g2 white calli were poor in carotenoids. Two lines (o298 and o306) had very low amounts of carotenoids (28 µg/g and 16 µg/g DW, respectively) while carotenoids were hardly detected in the remaining mutants (0.5–4.0 µg/g DW) (Figure 3E).

The expression of *psy2* gene was down-regulated (*p* = 0.003) in all psy2-g1g2 callus lines. It ranged from 0.19 to 0.49 with the mean of 0.29, relative to WT (Figure 3F,G). In the same callus, the expression of *psy1* was also down-regulated (*p* = 0.002) and ranged from 0.27 to 0.48 with the mean relative expression of 0.37. The expression of both genes highly correlated to each other (*r* = 0.97; *p* = 0.001). The carotenoids content also correlated with the expression of both genes, *psy2* (*r* = 0.93; *p* = 0.006) and *psy1* (*r* = 0.97; *p* = 0.001). The downregulation of *psy2*-edited lines may be also caused by NMD. The positive correlation between the expression of *psy1* and *psy2* may suggest also their coordinated regulation.

### 2.4. Altered Plastid Ultrastructure in the psy2 Mutants

**Figure 4:**
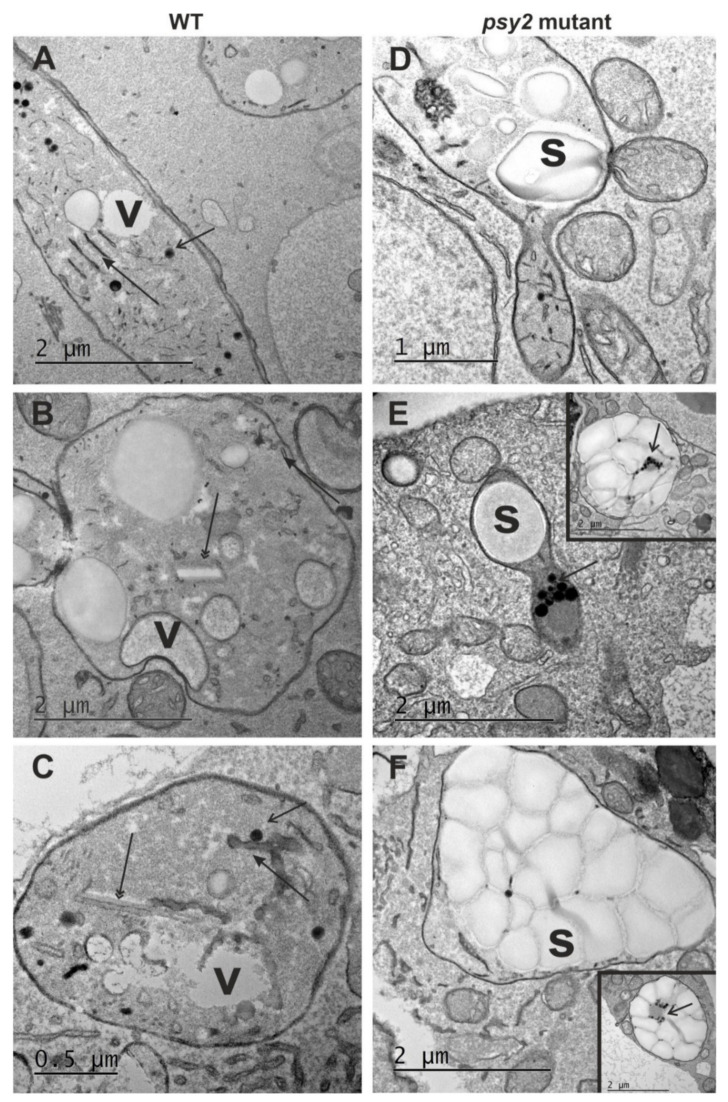
Plastid ultrastructure in callus cells of (**A**–**C**) the WT (not transformed control) and (**D**–**F**) *psy2* mutant. (**A**) Early stage of chromoplast differentiation in WT with plastoglobuli and internal membranes. (**B**) Chromoplast with a well visible carotene crystal. (**C**) Chromoplast with a carotene crystal, numerous plastoglobuli, internal membranes, and vacuoles. (**D**) Plastid in the *psy2* mutant callus cells with simple ultrastructure with the prominent starch grains, some plastoglobuli, and a few internal membranes. (**E**) Plastid with the single prominent starch grain and numerous plastoglobuli (inset–an example of amylochromoplast with numerous starch grains and plastoglobuli). (**F**) A plastid completely filled with starch grains surrounded by numerous mitochondria (inset–an example of plastid filled with the starch grains and numerous plastoglobuli). v-vacuole; s-starch grain; arrow-area with internal membranes; open arrow-plastoglobuli; double arrow-carotene crystal.

In non-green tissues, carotenoid accumulation and plastid biogenesis are interrelated, and carotenoids are commonly deposited in crystalloid or globular chromoplasts. To elucidate the effect of the impaired carotenoid biosynthesis on plastid ultrastructure, sections of the white *psy2* mutant and orange WT calli were examined to reveal differences at histological and ultrastructural levels. These callus lines differed in plastid ultrastructure (Figure 4). The plastids in WT callus cells had a structure typical for chromoplasts in the early stages of differentiation and they contained an abundant system of internal membranes, numerous plastoglobuli, and fine vacuoles (Figure 4A). In the later stages of differentiation, the internal membrane system was also clearly visible (Figure 4B). Carotenoid crystals were present in many plastids, which developed into chromoplasts classified to the crystalline type (Figure 4C). In the *psy2* mutant callus, amyloplasts were the most frequent plastid type; they had no internal membrane system and were almost completely filled with starch (Figure 4D–F). Some cells had plastids containing starch grains accompanied by plastoglobuli and small number of internal membranes, indicating an early stage of chromoplast differentiation (Figure 4D–F and insets). Unlike plastids, the cytoplasm ultrastructure of cells in both callus lines was similar. The cytoplasm was electron dense, with numerous mitochondria, vacuoles, abundant Golgi apparatus dictyosomes and endoplasmic reticulum membranes (Figure 4).

Both callus lines were composed of dividing and parenchymatic cells (Supplementary Materials, Figure S2A,B). WT callus consisted essentially of two types of cells. Dividing cells were almost isodiametric in shape and had dense cytoplasm strongly stained with toluidine blue O (TBO), and fine vacuoles (Supplementary Materials, Figure S2A). Many of these cells were at different stages of mitosis (Supplementary Materials, Figure S2C). WT callus also consisted of cells of parenchymatic character, which were oval and had large vacuoles (Supplementary Materials, Figure S2A). Callus of *psy2* mutant consisted of cells of the same two types, but the parenchymatic cells were less common than in WT callus (Supplementary Materials, Figure S2A,B). Regardless of callus line, all dividing cells were tightly attached to each other (Supplementary Materials, Figure S2C,D) and were distinguished based on the number of nuclei, numerous small vacuoles, and mitotic figures (Supplementary Materials, Figure S2C,D). There were two to three nuclei per cell (Supplementary Materials, Figure S2D).

### 2.5. Altered Cell Wall Composition in the psy2 Mutants

**Figure 5:**
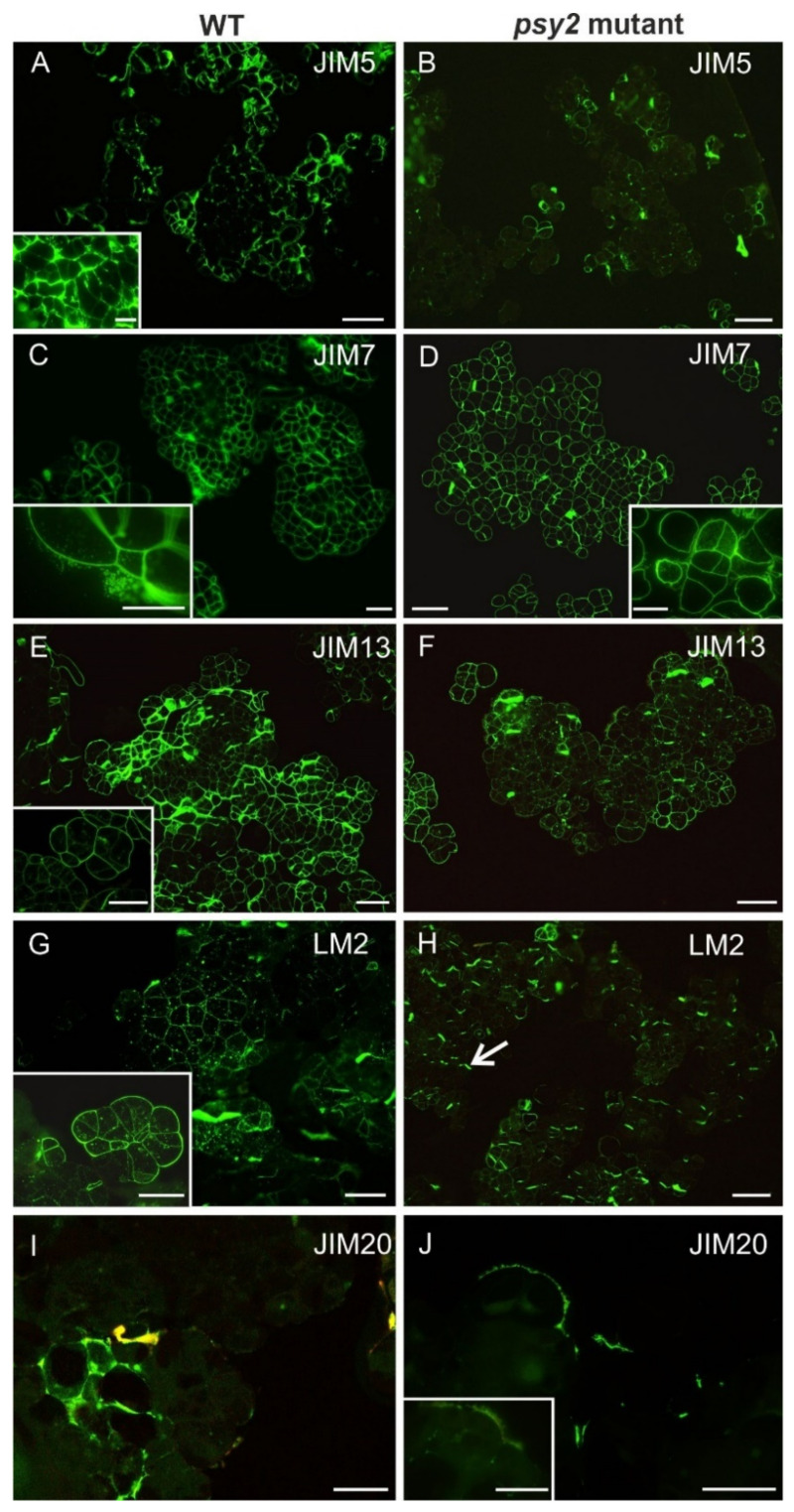
Distribution of pectic, AGPs, and extensin epitopes in callus cells of the WT (not transformed control) and *psy2* mutant. (**A**) The pectic epitope recognised by JIM5 antibody abundantly present in the cell walls (inset–higher magnification). (**B**) The presence of JIM5 epitope in the *psy2* mutant cells restricted only to a few cells. (**C**) JIM7 epitope present in all cells of WT (inset—higher magnification showing the presence of the epitope inside and outside the cells). (**D**) The distribution of JIM7 epitope in the *psy2* mutant cells (inset—higher magnification showing the presence of the epitope inside and outside the cells). (**E**) The presence of AGP epitope recognised by JIM13 antibody in WT cells. The epitope detected in the walls of all cells (inset—higher magnification showing the presence of the epitope also in the cytoplasmic compartments). (**F**) In *psy2* mutant cells the JIM13 epitope detected in much less amount in the cell walls in comparison to WT callus. (**G**) The AGP epitope recognised by LM2 antibody present in the walls of all cells and in the cytoplasmic compartments of WT callus (inset). (**H**) In *psy2* mutant cells this epitope detected mainly in new walls (arrow). (**I**) The extensin epitope recognised by JIM20 antibody was present only in the walls of few cells in WT callus. (**J**) In *psy2* mutant cells this epitope detected also in some cells, within the walls but also outside the walls (inset). Scale bars: (**A**–**H**) 200 µm; (I, J) 100 µm; insets 100 µm.

To reveal whether impairing carotenoid biosynthesis may induce changes in the cell wall composition, AGPs, pectins and extensin spatial distributions in callus cells were assessed. For this purpose, immunohistochemical staining was applied to reveal differences in the cell wall composition between the *psy2* mutant and WT. Low-methylesterified (detected by JIM5 antibody) and highly methylesterified (detected by JIM7 antibody) homogalacturonan (HG, pectins) were present in the cell wall of both callus lines (Figure 5; Supplementary Materials Table S1). The distribution patterns of de-esterified and esterified pectins in cell walls were different and corresponded to the carotenoid contents. Low-methylesterified pectins were abundantly present in walls of each cell in carotenoid-rich WT callus (Figure 5A) but in the white *psy2* mutant they were detected only in some cells (Figure 5B; Supplementary Materials Table S1). In contrast, fluorescence signals coming from highly esterified pectins had similar intensities in WT and mutant calli (Figure 5C,D). Additionally, in the WT callus the JIM7 epitope was present also outside the walls and in cytoplasmic compartments (Figure 5C and inset). The AGP epitopes, recognised by the JIM13 and LM2 antibodies, were abundant in WT callus while their presence in the *psy2* mutant was low (Figure 5E,H; Supplementary Materials Table S1). The extensin epitope recognised by the JIM20 antibody was not abundant, however, it was present in the walls of more cells in the *psy2* mutant than in WT (Figure 5I,J; Supplementary Materials Table S1).

## 3. Discussion

To reveal the roles of *psy* paralogs and their effect on the accumulation of carotenoids and metabolites involved in the cell wall composition, two sets of model callus lines with mutations at either *psy1* or *psy2* have been developed in this work using CRISPR/Cas9, which has been extensively utilized for site-directed mutagenesis [37,38]. *Psy* gene knockouts were previously obtained using CRISPR/Cas in other species. In tomato, the *psy1* knockout mutants had reduced accumulation of carotenoids in petals and fruits [39,40]. In maize, *psy* mutations led to the development of albino plants and formation of white seeds [41]. In carrot, the carotenoid pathway genes were targeted using CRISPR/Cas9 [42]. The pioneering work showing CRISPR/Cas-mediated gene editing in carrot was done using callus as a model and the flavanone 3-hydroxylase gene in anthocyanin biosynthesis pathway was edited [43]. The functional mutants had altered pigmentation, including complete discolouration, thus they were identified visually. The same approach has been applied in this work where carotenoid-rich callus distinguished by a dark orange colour was used. The observed colour changes thus indicated putative *psy* knockout due to downregulation of the carotenoid pigments biosynthesis. As expected, several small callus clumps developing on the explant surfaces were light orange and those further developed on psy2-g1g2 calli were white. Visual assessment revealed that discoloured cell foci developed in prolonged culture of orange callus, indicating the occurrence of de novo mutations in non-mutated cells. Such effects of delayed editing could appear due to constitutive expression of Cas9 and gRNAs [44]. White foci were observed only for psy2-g1g2 callus, which was a visible sign of the completely inhibited accumulation of carotenoids that could result from knockout of the carotenoid pathway key genes as shown earlier for a phytoene desaturase knockout [45]. To increase the frequency of potential mutations, we introduced to callus cells two gRNAs targeting the same gene at different locations. Upon successful transformation, various *psy* mutations were identified. They occurred at one of the two gRNA target sites for each gene or at both sites, generating deletions of long DNA fragments spanning between the targets. Several methods are used for the identification of Cas-induced mutants [46]. In the first method, loss of a restriction enzyme site due to Cas9-induced mutations was used. This method, however, could not capture all targeted mutations. Hence, the T7E1 assay was used as an alternative. The T7EI assay however may remain insensitive to long deletions, does not recognise homoduplexes at mutant sites and does not reveal the nature of editing event. Thus, this assay is considered as complementary to other methods to increase the chance for detection of mutants, which were eventually revealed by Sanger sequencing [47]. Hence, the size polymorphism of *psy* restriction fragments obtained after using specific endonucleases accompanied by the T7EI assay and, finally, sequencing, collectively confirmed that the selected callus lines here contained cells with edited *psy* genes.

Long deletions spanning both upstream and downstream the *psy1* and *psy2* target sites were found while the deletion of the whole fragment (−286 nt) between gRNA1 and gRNA2 targets occurred only in the *psy2* mutant lines, probably because of a small distance between targets (<300 bp), which enables such deletions when DSBs at both sites occur simultaneously [48]. The whole fragment duplication (+286 nt) was also found as a rare event. Cas9-induced long fragment insertions and inversions have been reported [42,49]. Most of the mutations detected here resulted in the reading frame shift and the occurrence of premature stop codons. Even a single amino acid modification may affect PSY activity as it was shown in cassava where the A191D amino acid exchange increased 20-fold the carotenoid content in the root [50]. According to the InterPro database [51], the trans-isoprenyl diphosphate synthases active domain (IPR033904) between 112–379 amino acids in the carrot PSY1 has two conserved sites at 229–244 aa and 265–290 aa. In PSY2, this domain (146–413 aa) has the conserved sites at 263–278 aa and 299–324 aa. Thus, in this work, the alterations generated in the amino acid sequence at about 17 aa and 250 aa in PSY1, and, at about 39 aa and 135 aa in PSY2 occurred either upstream or within the conserved domains, which would considerably affect the structures of PSY proteins. In particular, the presence of premature stop codons most likely led to the synthesis of truncated, non-functional proteins. Additionally, the mutants resulted in decreased expression of *psy*1 or *psy2* genes by 50−80%, with the mean reduction by 71% in comparison to WT, independent on the target gene. In consequence, the carotenoid contents were at low or only hardly detectable levels, which unambiguously indicates functional mutations, except for two *psy1* lines (o186 and o196).

The lack of carotenoids in the *psy2* mutants did not correspond with 30% transcription level, but this phenomenon can be explained by a feedback regulation. Changes in the expression levels of genes downstream the carotenoid pathway could affect *psy* expression [52]. The PSY enzyme levels may also change even when the *psy* transcript levels are not altered, indicating that the protein level is modulated by carotenoid metabolites and their precursors [53,54]. In Arabidopsis grown in the dark and in tomato, an increased PSY activity promoted post-transcriptional accumulation and activity of 1-deoxy-D-xylulose 5-phosphate synthase (DXS), the main rate-determining enzyme controlling the MEP pathway flux [53,55]. Hence, PSY activity regulates the availability of MEP pathway-derived precursors. On the other hand, it was shown that overexpression of DXS upregulates *psy1* and *psy2* expressions in carrot leaves and increases carotenoid levels in the roots of dark-grown plants [56]. Hence, the decreased *psy* transcription found in the carrot mutants in this work might be regulated by a lowered DXS level or activity. In the *psy2* mutants, the *psy2* expression decreased, so did the *psy1*, despite that psy2-gRNAs did not target the *psy1* gene. Thus, the knockout of *psy2* reduced the expression of the other paralog, which could be realised by the feedback regulation indicated above. This mechanism is, however, not equally efficient for both paralogs as in some *psy1* mutants the carotenoid level remained unaffected. Moreover, regardless of which gene was targeted, the carotenoids content correlated with the *psy2* transcript level and all *psy2* mutants did not accumulate carotenoids. Hence, *psy1* did not compensate for the absence of functional PSY2. In contrast, *psy1* mutants showed variation in carotenoid contents and only those which had high *psy2* expression were able to accumulate carotenoids in high amounts. All these suggest that the role of PSY1 can be only accessory. These results show that PSY2, but not PSY1, is critical for carotenoid biosynthesis in this callus system. Analogous relationships were shown in pepper fruits where *psy2* compensated for the absence of *psy1*, which is fruit specific in pepper [57]. The lack of carotenoids in the *psy2* mutants may also suggest that either all PSY were non-functional or post-transcriptional regulation was affected that is congruent with previous observations showing similar *psy* transcript levels in both orange and white carrot roots [29]. This can be also related to the development of chromoplasts triggered by the PSY and Orange (OR) protein interaction, also in callus [30,58,59]. A strong post-transcriptional co-regulation between PSY and OR exists in Arabidopsis as PSY overexpression increases OR level while PSY suppression decreases OR level. Additionally, OR overexpression results in higher level of active PSY protein and carotenoid content but not *psy* transcript level [60].

Carotenoid accumulation is interdependent on plastid biogenesis [5] and in the carrot storage root these compounds are synthesised and sequestered in chromoplasts. Mainly crystalloid chromoplasts were found in carrot [61,62] but small number of globular chromoplasts containing numerous plastoglobuli, with usually lipid-dissolved carotenoids, were also identified [63]. Chromoplasts develop as the result of initial proplastids differentiation to leucoplasts and amyloplasts. The latter is indicated as chromoplasts precursors [64] and, at early stages, such amylochromoplasts still contain small starch grains accompanied by globular and crystalline structures containing carotenoids [65]. In the carrots developing white roots and in young roots of orange carrots large amyloplasts were abundant while in fully developed storage orange roots amyloplasts were not observed [61]. Carrot callus which usually is poor in carotenoids also contains many amyloplasts. In this work, the dark-orange callus with enhanced carotenoid biosynthesis was used, which has additional amylochromoplasts and crystaloid chromoplasts that are the dominating plastid type [34]. Hence, a transition of amyloplast to chromoplasts has been proposed as a hypothetical route during storage root development. The ultrastructure analysis of WT and the *psy2* mutant callus lines in this work provides further evidence supporting this hypothesis as the disturbance in the carotenoid metabolic pathway induced ultrastructural changes of chromoplasts. In WT callus, the chromoplasts were abundant and were represented mostly by the crystalline type as described earlier for this callus line [34] and their ultrastructure had typical organisation, like the one described for carrot root [22,66]. In contrast, in the *psy2* mutant cells the starch-filled plastids were found instead of chromoplasts. Therefore, these cells contained large amyloplasts similar to the cells in the white carrot root [61]. Only some cells had plastids with fragmented internal membranes and a few plastoglobuli coexisting with starch grains. Such organisation indicates an early stage of chromoplast differentiation. The *psy2* mutant cells had no visible pigmentation but the *psy* gene expression occurred at a low level and small amounts of carotenoids were found. The observations of the ongoing transition of amyloplasts to amylochromoplasts in some cells additionally supports the expected plastid differentiation route as the requirement for carotenoids biosynthesis and further sequestration. However, the lack of later stages of chromoplast development indicates that the amounts of carotenoids were insufficient to complete amylochromoplast transition. The presence of plastoglobuli may indicate that at first carotenoids are bound to lipids, and crystals are sequestered only when there is an excess of carotenoids. Such interpretation is in accordance with results presented earlier [34]. It is worth noting that the ultrastructure of plastids in the carotenoid-rich WT dark orange callus resembles the ultrastructure of plastids in orange carrot roots [34] while the ultrastructure of plastids in the carotenoid-free (carotenoid-poor) *psy2* mutant callus resembles the ultrastructure of plastids in white carrot roots [61] where carotenoid biosynthesis pathway is non-functional. Therefore, developed callus seems a valuable model system for further elucidation of carotenogenesis and plastid biogenesis, and their regulation, and their effects on other biochemical processes.

The biosynthesis of carotenoids and polysaccharides depends on the same carbon source but their interrelations are more complex. Changes in the *psy* activity may induce modifications in the cell metabolism. In tobacco, the silencing of *psy1* and *psy2* genes not only markedly decreased the carotenoid biosynthesis but also affected pathways that are involved mainly in the biosynthesis of cell wall components, and the expression of glucan, cellulose, pectin, and galacturonan genes [11]. Suppression of carotenoid metabolism in tomato led to downregulation of genes encoding major cell wall catabolic enzymes and increased fruit firmness [67]. Overexpression of the *SlNAC1* transcription factor in tomato lowered lycopene and total carotenoid contents and influenced cell wall enzymes leading to the increased fruit softening during their ripening. Hence, it was postulated that cell wall components were metabolised via an ABA-dependent pathway [68]. Additionally, the overexpression of *LCYb* gene in tomato resulted in the enhanced metabolism of lycopene to β-carotene and in the increase of ABA content what was linked with extended fruit shelf life [9]. Gene ontology analysis revealed that several genes involved in cell wall organisation and hydrolase activity of *O*-glycosyl compounds were upregulated also in soybean containing the insertion of *psy* overexpressing construct [10]. The *yellow-fruited tomato 1* (*yft1*) mutant, which fruit contained only 6% of the total carotenoids of that of the wild type cultivar, had delayed chromoplast development and a modified cell wall composition [69,70]. Such co-occurring changes in *yft1* resulted from a mutation in an ethylene-insensitive 2 protein, a core factor for the ethylene signal transduction connecting these processes and carotenoid accumulation [71] but there was no evidence for a direct link between carotenoid biosynthesis and wall composition. A negative correlation was noted between the cell wall thickness and total carotenoid content in segment membranes and sac membranes in grapefruit [72]. Additionally, the fungal cell wall was modified when exposed to carrot extract, in particular lutein induced α-1,3-glucan accumulation [73]. These reports collectively indicate that regulation of carotenoid biosynthesis may affect structural changes in the cell wall although the spatial distribution of wall components in relation to the carotenoid biosynthesis has not been shown so far. In this work, immunohistological data show alterations in the wall composition. Apparently, the differences in the presence of chosen pectic, AGPs, and extensin epitopes occurred between the carotenoid-rich control and the carotenoid-free *psy2* knockout mutant. Among analysed pectic epitopes, low-esterified pectins were less represented in the walls of the *psy2* mutant cells in comparison to WT, but the presence of highly esterified pectins was not affected by the *psy2* gene knockout. This may mean that the changes in carotenoids synthesis affect the degree of pectin de-esterification; however, the signalling pathway between the carotenoid synthesis and pectin modifying enzymes is not yet known. Pectins are the major group of primary wall polymers [74,75] responsible for wall porosity, adhesion, binding of ions, and mechanical properties of cell walls, including elasticity [75]. Pectin methylesterase activity can increase or decrease the degree of calcium-cross linking of homogalacturonan molecules, thus modifying the wall stiffness in different degree [76,77]. Since the inhibition of carotenoid biosynthesis restricts pectin de-esterification, it can be concluded that the lower degree of highly esterified pectins may indicate the reduced cell wall stiffness as described for various plant organs [77,78]. Moreover, the increased level of pectins with a low degree of methylation was detected in *LCYb*-overexpressing tomato fruits, indicating that their increased level was associated with enhanced carotene biosynthesis [9]. The presented results, showing the relationship between carotenoid synthesis and the pectins modifications, are in agreement with the widely accepted opinion that changes in the methylesterification status of pectins are related to the cell wall remodelling that occurs during diverse plant developmental processes and in reaction to biotic and abiotic factors [79,80].

Arabinogalactan proteins (AGPs) belong to subfamily of hydroxyproline-rich glycoproteins (HRGPs; [81,82]). They are believed to be present in each plant cell including cellular compartments such as wall, plasma membrane, and membranes inside of the cell [83,84]. Involvement of AGPs in many developmental processes, such as signal transduction pathways, cell growth and differentiation, programmed cell death, and hormone responses are well documented [85,86,87]. Moreover, AGPs are believed to be involved in the plant response to a wide range of biotic and abiotic stresses [88,89]. Spatial distribution of analysed AGPs epitopes in this work suggests that knocking out the *psy2* gene considerably affected the presence of LM2 and JIM13 epitopes. Such results are in accordance with the literature data indicating that changes in AGP presence are the manifestation of reaction of cells to changes in cell metabolism. The LM2 antibody detects the AGPs epitope that contains glucuronic acid (GlcA) residues [90]. Our research shows that the LM2 epitope is less represented in the walls of cells from the *psy2* mutant. Analogous occurrence was detected for the JIM13 epitope (which contains β-D-GlcpA-(1→3)-α-D-GalpA-(1→2)-L-Rha residues). This indicates that, at least for these two AGP epitopes, the blocking of carotenoid biosynthesis results in the decrease of AGPs synthesis. Undoubtedly, impaired biosynthesis of carotenoids is a stress factor for the cell. Considering that changes in AGPs contents are the cell response to biotic and abiotic factors [83], the obtained results here are not surprising. However, we showed for the first time that alterations in carotenoid biosynthesis are related to cell wall remodelling, which was visualised directly in the cell walls with the use of immunohistochemistry method. Furthermore, some changes in gene expression and protein synthesis in soybean that were connected to carotenoid biosynthesis were detected for fascilin-like AGP [10]. Our study presents another data that indicate a correlation between AGPs presence and carotene biosynthesis.

Extensins are another group of hydroxyproline-rich glycoproteins, which are structural cell wall proteins characterised by the presence of Ser-Pro2–n repeats (for review see [91]). These proteins are common in different species (for review see [92]), including carrot [93,94]. Extensins can occur not only in the cell wall matrix, but also in intercellular spaces [92]. Recently, they have been found in extracellular layer covering callus cells [95]. In the present work, the JIM20 extensin epitope was found in callus cells of both, the control and the *psy2* mutant, but only in a few cells. The only difference between the control and mutant was the spatial JIM20 distribution. In the control the extensin epitopes were detected in the walls, and in the mutant the epitopes were present in the extracellular matrix that covered cell aggregates. Callus of the control and the *psy2* mutants differed not only in terms of cell types but also in deposition of extracellular material on the outer walls of peripheral callus cells, which was more pronounced in mutant. The occurrence of extensins in extracellular material may point to different callus competence, what was found for *Actinidia arguta* [96]. Moreover, the involvement of extensins in the development of carrot protoplast-derived cells was also described [97].

## 4. Materials and Methods

### 4.1. Plant Material

Callus obtained from roots of the DH1 plants described earlier and characterised by a dark-orange colour due to high accumulation of carotenoids was used [34]. Callus was maintained in Petri dishes on the BI medium (Gamborg B5 mineral medium with vitamins, supplemented with 1 mg/L 2,4-dichlorophenoxyacetic acid, 0.0215 mg/L kinetin and 30 g/L sucrose; pH 5.8; solidified with 2.7% Phytagel) at 26 °C in the dark and was subcultured every 3 weeks to a fresh medium.

### 4.2. Vector Construction

Two pairs of gRNAs were designed to target the *psy1* (GeneBank Gene ID: 108227339) and *psy2* (GeneBank Gene ID: 108214656) genes based on a reference ASM162521v1 primary assembly of the DH1 carrot genome. Each gRNA was verified not to target another *psy* paralog and other genes in the DH1 carrot genome using the Cas-OFFinder online tool [98]) with default settings. Two plasmid vectors (psy1-g1g2 and psy2-g1g2) were created (Figure 1A,B); each of them contained in the T-DNA region a pair of gRNAs under the control of AtU3 (gRNA1) and AtU6 (gRNA2) promoters, the *Cas9* gene driven by the double 35S CaMV promoter, and the *aph* hygromycin resistance gene also driven by the double 35S promoter. The psy1-g1g2 vector contained gRNA1 (psy1-g1: TATTATCCCAAAGAGATTCATG) complementary to exon 1 in the carrot *psy1* gene and gRNA2 (psy1-g2: GTGGTGGCCTTTGATTCCG) complementary to exon 4 (Figure 1A). The psy2-g1g2 vector contained two gRNAs complementary to exon 1 of the *psy2* gene, gRNA1 (psy2-g1: ATCCATCTAGGTTGGGTTCCC) and gRNA2 (psy2-g2: GCCAGAAATGATTCTTCCG) (Figure 1B). All gRNAs were designed to target sites adjacent to GGG-PAM located either upstream (gRNA1) or within (gRNA2) the region coding for the protein active domain (Figure 1C). Vectors were assembled according to [99] and introduced into *Agrobacterium tumefaciens* LBA4404 strain by electroporation [100].

### 4.3. Agrobacterium-Mediated Callus Transformation

*Agrobacterium*-mediated transformation of carrot callus was done as described previously [43]. Briefly, callus clumps of about 5 mm in diameter were immersed for 20 min in *A. tumefaciens* inoculum composed of the BI medium supplemented with 100 µM acetosyringone with OD_600_ adjusted to 0.5. After three days of co-cultivation, callus was rinsed with 800 mg/L cefotaxime and 400 mg/L timentin mixture and cultured on the BI medium supplemented with these antibiotics at concentrations lowered to a half. Putative transformants were selected after three-month culture on the BI medium supplemented with 25 mg/L hygromycin.

### 4.4. Molecular Identification of Mutants

Genomic DNA extraction, PCR, digestion of amplified fragments with restriction enzymes, cloning and sequencing were performed essentially as described previously [43]. To confirm the presence of T-DNA in putatively transgenic callus, PCR primers matching *aph* and *Cas9* genes were used while the amplification of *psy* gene fragments was obtained using primer pairs flanking either single gRNA targets in the *psy1* gene or both gRNA target sites in the *psy2* gene (Supplementary Materials, Table S2). To detect mutations at 1−4 nucleotides upstream PAM within gRNA targets, the amplified *psy* gene fragments were digested using specific endonucleases recognising restriction sites at these sites (Figure 1A,B). Additionally, the T7 endonuclease I (T7EI) assay was performed to detect mutations that might occur outside restriction sites recognised by the above said enzymes. For this purpose, 4 µL of PCR reaction obtained using Phusion™ High–Fidelity DNA Polymerase (ThermoScientific, Waltham, MA, USA) were incubated with 0.3 µl T7 endonuclease I (New Englands BioLabs, Ipswich, MA, USA) at 37 °C for 25 min, following hybridization according to manufacturer’s instruction. The reaction was stopped by the addition of 1.5 µl of 0.25 M EDTA. Products of PCR and cleavage were visualised after electrophoresis in a 2% agarose gel containing MidoriGreen Advance (Nippon Genetics, Tokyo, Japan). Undigested fragments by specific endonucleases were purified and cloned (5−7 clones per callus line) before Sanger sequencing, for which the Sp6 standard primer was used. Reads were manually aligned to the reference gene sequences.

### 4.5. Determination of Carotenoids Content

The whole plate of freshly grown callus was lyophilized and ground in a mortar. Extraction was done using ethanol:*n*-hexane (1:1, *v:v*). Extracts were filtered through a sintered glass funnel (G4) before analysis. A high-performance liquid chromatography (HPLC) was performed using the Shimadzu LC–20AD chromatograph equipped with a C18 RP (5 µm) column and the Shimadzu SPDM–20A–DAD photodiode–array detector. The signal detection was set in the wavelengths range of 300–700 nm. The separation was carried out at 25 ± 1 °C using the solvents: (A) 5% water in methanol, (B) methanol, (C) 10% *n*-hexane in acetonitrile). All solvents were ultra pure (Sigma–Aldrich, St. Louis, MO, USA). The identification of β-carotene was based on the retention time of the standard (Sigma–Aldrich, St. Louis, MO, USA) and confirmed by analysis of absorption spectra. The identification of α-carotene and xanthophylls was based on the analysis of the absorption spectra. Quantification of β-carotene was done using a standard curve, while α-carotene and xanthophylls were quantified in relation to β-carotene. The analysis was performed in three biological replicates for each callus line.

### 4.6. Gene Expression Analysis

RNA isolation and gene expression analysis was performed using a real-time quantitative PCR as described previously [34]. Primers were designed to amplify fragments of exon 5 and exon 4 in *psy1* and *psy2* genes, respectively (Supplementary Materials, Table S2). Normalization was done to the expression of the actin gene. Gene expression analysis was performed for the control and each of ten selected mutant lines, using mixed samples from five callus clumps.

### 4.7. Light Microscopy

For the histochemical analyses, the samples were fixed in a solution of 3% (*w/v*) paraformaldehyde (PFA), 1% (*v/v*) glutaraldehyde (GA) and 1% sucrose (*w/v*) in phosphate buffered saline (PBS) at pH 7.0, embedded in Steedman’s wax [95]. The sections (7-μm thick) were cut using a HYRAX M40 rotary microtome (Zeiss, Oberkochen, Germany) and collected on microscopic slides. Sections were stained with 0.1% toluidine blue O (TBO; Sigma-Aldrich, St. Louis, MO, USA) in PBS and examined under the Olympus BX45 microscope equipped with the Olympus XC50 digital camera.

### 4.8. Fluorescence Microscopy

For immunohistochemistry, samples were proceeded as described earlier [101]. Briefly, samples were fixed in a mixture of 4% formaldehyde and 1% glutaraldehyde (pH 7.2) at 4 °C for 24 h, then washed in PBS (pH 7.2), dehydrated in a graded ethanol series, infiltrated in LR White resin (medium grade, Polysciences, Eppelheim, Germany). Finally, they were embedded in gelatine capsules with fresh LR White resin and polymerized at 50 °C for 8 h. Semi-thin sections (0.5–1 μm) were cut using the Leica EM UC6 ultramicrotome (Leica Microsystems, Wetzlar, Germany) and mounted on poly-l-lysine coated microscope slides (Menzel-Glaser, Braunschweig, Germany). Primary antibodies used in the study are listed in Table 1.

### 4.9. Transmission Electron Microscopy (TEM)

Samples were fixed in 3% glutaraldehyde in a 50 mM cacodylate buffer (Serva, Heidelberg, Germany; pH 7.0) at 4 °C for 24 h. Then, samples were washed three times in cacodylate buffer and post-fixed with 1:1 (*v*/*v*) mixture of 3% potassium ferrocyanide in cacodylate buffer and 4% solution of osmium tetroxide (Serva, Heidelberg, Germany) for 1 h. Samples were washed three times in dH_2_O and incubated in 1% solution of thiocarbohydrazide (Sigma-Aldrich, St. Louis, MO, USA) at 60 °C for 20 min. Next, samples were post-fixated in 1% aqueous osmium tetraoxide at room temperature for 30 min, rinsed three times in dH_2_O, and incubated overnight in 1% aqueous uranyl acetate at 4 °C. The samples were then rinsed three times (each 5 min) in dH_2_O and put into a freshly prepared Walton’s lead aspartate for 30 min at 60 °C, washed five times (each 3 min) in dH_2_O, dehydrated in graded ethanol series, and in the mixture of 99.8% ethanol and propylene oxide (1:1 *v*/*v*, 15 min), and propylene oxide (2 × 15 min) and gradually embedded in Epon resin (Poly/Bed 812; Polysciences, Eppelheim, Germany). Ultrathin sections of 70 nm thick were cut with the use of the Leica EM UC6 ultramicrotome and collected onto carbon-coated copper grids (200 mesh, Electron Microscopy Science, Hatfield, PA, USA). Samples were analysed in the Jeol JEM-3010 HRTEM (300 kV) equipped with an EDS (Energy Dispersive Spectrometry, IXRF Systems Inc., Austin, TX, USA) spectrometer and a 2 k × 2 k Orius 833 SC200D CCD camera (Gatan, Pleasanton, CA, USA).

### 4.10. Statistical Analysis

The effect of *psy1* and *psy2* gene mutations on carotenoid content in callus was verified by applying one-way analysis of variance followed by the multiple comparison Tukey’s test at the significance level *p* = 0.05. Person linear correlation was calculated to reveal relationship between gene expression and carotenoids content. Data were analysed using the Statistica v13 software (TIBCO; Palo Alto, CA, USA). Relative gene expression was calculated using the REST 2009 (Qiagen, Hilden, Germany) software.

## 5. Conclusions

In this work, we have successfully knocked out two carrot *psy* genes using CRISPR/Cas9 system and studied the effect of the induced mutations on carotenoids accumulation, plastid ultrastructure, and the cell wall composition. We used callus as a model system that is characterised by high carotenoid content and gene expression levels resembling those observed in the carrot root. The results indicate differential roles and regulation of *psy* paralogs, with *psy2* being critical for carotenoid biosynthesis in this model. Moreover, *psy2* knockout reduced the expression of the *psy1* paralog, which could be explained by the metabolite feedback regulation. Whether, or to what extent, *psy1* contributes to carotenogenesis in callus remains unclear as there was no correlation between its expression level and carotenoid content when *psy2* was active. To the best of our knowledge, it was also demonstrated for the first time that the impaired biosynthesis of carotenoids interrelates with cell wall remodelling, which is manifested by changes in the composition of pectins and AGPs within the wall. Thus, this work presents that a relationship exists between the cell wall and carotenoid metabolisms, and that the dark orange callus may serve as a usable model for basic genomic studies as well as cell metabolism related to carotenogenesis. Further research should be commenced to reveal these relationships in fully developed plants.

## Figures and Tables

**Table 1:** Primary antibodies used for the detection of cell wall components.

Wall Constituents	Antibody	Epitope	References
Pectins	JIM5	Low methyl-esterified HG	[102]
	JIM7	Highly methyl-esterified HG	[102]
AGPs	LM2	β-d-GlcpA	[90] [103]
	JIM13	β-d-GlcpA-(1→3)-α-d-GalpA-(1→2)-l-Rha	[104] [103]
Extensins	JIM20	Extensin/HRGP glycoprotein	[105]

Abbreviations: HG—homogalacturonan, GlcA—glucuronic acid, GalA—galacturonic acid, Rha—rhamnose, HRGP—hydroxyproline-rich.

## Data Availability

Not applicable.

## Supplementary Materials

The following are available online at https://www.mdpi.com/article/10.3390/ijms22126516/s1, Figure S1: Amino acid sequences of PSY in the wild type and mutant lines, Figure S2: Morphology and structure of the wild type and *psy2* mutant callus, Table S1: Primers used for PCR and qPCR, Table S2: Cellular epitopes localization and degree of their occurrence within the callus of different lines.
