# Supplementary material for: Inhibition of Carotenoid Biosynthesis by CRISPR/Cas9 Triggers Cell Wall Remodelling in Carrot
Source: Int J Mol Sci. 2021 Jun 17;22(12):6516. doi: 10.3390/ijms22126516 (PMC8234013; doi:10.3390/ijms22126516)
Supplement: Supplementary file 1 [file ijms-22-06516-s001.zip › ijms-1217316-supplementary.pdf]

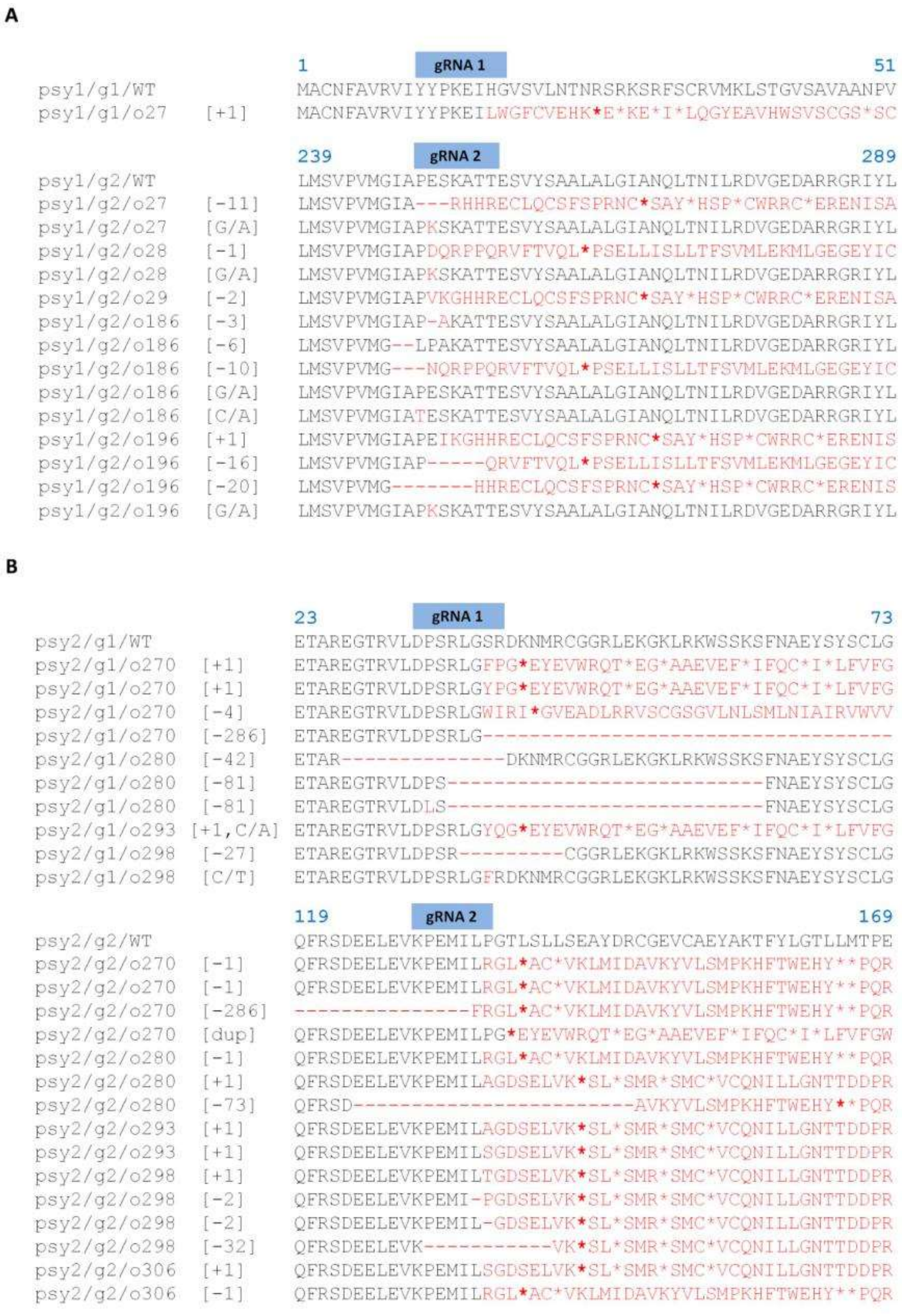

Figure S1 Amino acid sequences of PSY in the wild type (WT; not transformed control) and mutant lines. The sequence changes in mutants in comparison to WT are marked in red. (**A)** Sequences of PSY1 in psy1-g1g2 lines. (**B)** Sequences of PSY2 in psy2-g1g2 lines.

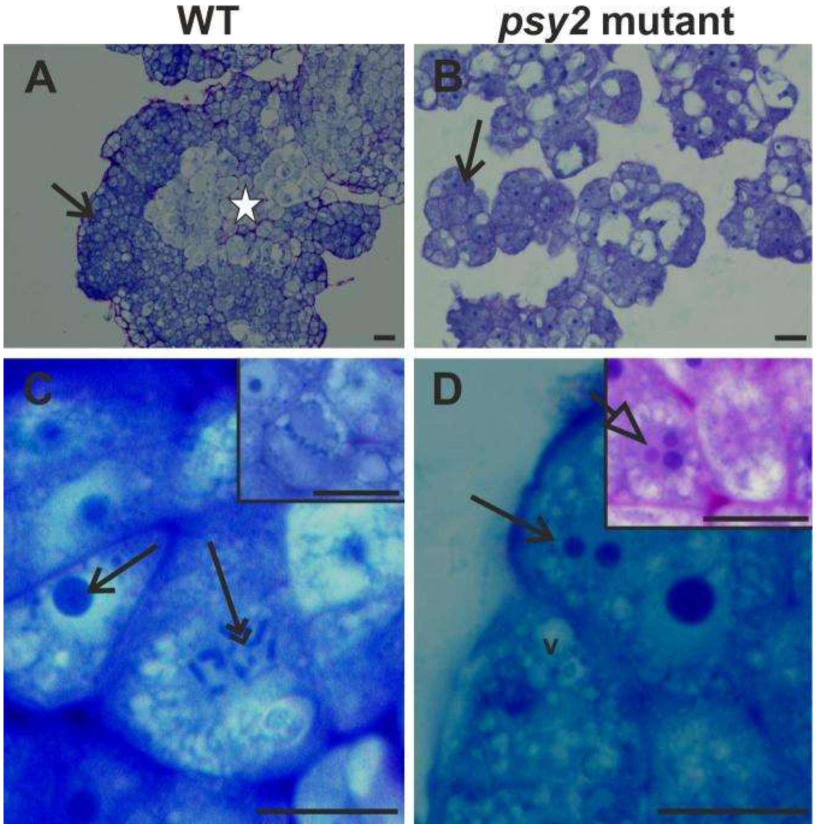

Figure S2 Morphology and structure of (**A** and **C**) the wild type (WT; not transformed control) and (**B** and **D**) *psy2* mutant callus. (**A**) Dividing cells (arrow) localized at the periphery of callus clumps surrounding the parenchymatic cells (white star). (**B**) *psy2* mutant callus composed mainly of dividing cells (arrow) characterised by dense cytoplasm.

(**C**) The dividing WT cells were characterised by large nucleus with prominent nucleolus (arrow), and numerous cells in different phases of mitosis (double arrow; inset – metaphase plate). (**D**) *psy2* mutant callus cells with different number of nuclei (two nuclei – arrow; three nuclei – inset, empty arrow) and numerous small vacuoles (v). Scale bars = 20 µm.

Table S1 Primers used for PCR and qPCR

Amplified fragment	Primer name	Primer sequence 5' - 3'
aph	aph-F	AAGGAATCGGTCAATACACTACATGG
	aph-R	AAGACCAATGCGGAGCATATACG
35S:*Cas9*	CaMV-35S-F	CCACGTCTTCAAAGCAAGTGG
	AteCas9-R	TTGGGTGTCTCTCGTGCTTC
psy1 (gRNA1 region)	psy1-F1	ACAACCCAACTCAACTCTCTCT
	psy1-R1	TACTCAGCACAAACTTCGCCA
psy1 (gRNA2 region)	psy1-F2	CCTCCCATATCACGCCCAAG
	psy1-R2	ACTCTTCTCCAACATCACGG
psy2 (gRNA1+gRNA2)	psy2-F	GTTGGTGTGAATGGTTGAGGC
	psy2-R	AGTAATTTAGAGGCAGTCAGCA
psy1 (qPCR)	psy1-qpcr-F	GGGAATAACACCAGAATAC
	psy1-qpcr-R	ATCTACTTGCTGAACTTAAC
psy2 (qPCR)	psy2-qpcr-F	TATGTTGCTGGTACTGTTGG
	psy2-qpcr-R	CGTTGTTGCCTGTGAATTAG

Table S2

Cellular epitopes localization and degree of their occurrence within the callus of different lines

Epitope	WT	*psy2* mutant
**pectins**		
JIM5	walls, intercellular spaces<br>**+++**	walls, cytoplasmic compartments<br>**+**
JIM7	walls, cytoplasmic compartments and secreted outside the walls of peripheral cells<br>**+++**	walls, cytoplasmic compartments and secreted outside the walls of peripheral cells<br>**+++**
**AGPs**		
JIM13	walls,<br>cytoplasmic compartments<br>**+++**	walls,<br>cytoplasmic compartments in selected cells<br>**+**
LM2	walls,<br>cytoplasmic compartments<br>**+**	present in new cell walls,<br>cytoplasmic compartments in some cells<br>**+/-**
**extensin**		
JIM20	walls, intercellular spaces<br>**-**	extracellular location<br>**+**

The percentage of cells that contain the epitope was taken into account: − no labelling, indicates the lack of epitope or the presence below 1% of cells, +/- weak labelling indicates the presence of the epitope between 2-10% of callus cells, + moderate labelling indicates the presence of epitope between 20-40% of callus cells, and +++ 100% callus cells expressing the epitope; the values are derived from three samples of each type of callus.
